# Use of a Novel, Reinforced, Low Immunogenic, Porcine Small Intestine Submucosa Patch to Repair a Supraspinatus Tendon Defect in a Rabbit Model

**DOI:** 10.1155/2019/9346567

**Published:** 2019-04-03

**Authors:** Xuancheng Zhang, Zhaoyi Fang, Eunshinae Cho, Kai Huang, Jinzhong Zhao, Jia Jiang, Xiaoqiao Huangfu

**Affiliations:** Department of Sports Medicine, Shanghai Jiao Tong University Affiliated Sixth People's Hospital, 600 Yishan Road, Shanghai 200233, China

## Abstract

**Background:**

Repairs of large to massive rotator cuff tears have a high failure rate. We investigated the efficacy of a novel, reinforced, low immunogenic, porcine small intestine submucosa (SIS) patch to repair a supraspinatus tendon defect in a rabbit model. We hypothesized that the histological and biomechanical results of SIS patch repair would be comparable with those of autologous fascia lata (FL) repair.

**Methods:**

The study mainly comprised two parts. First, the characteristics of the SIS patch were evaluated, including its micromorphology, mechanical properties, and immunogenic properties. Second, a supraspinatus tendon defect model was created in 36 rabbits (72 shoulders). The bilateral shoulders were randomly chosen to undergo repair using either a SIS patch (SIS group) or autologous FL (FL group). At 4, 8, and 12 weeks, histological analysis was performed using four shoulders from each group, and biomechanical tests were performed using eight shoulders from each group.

**Results:**

The SIS patch was a three-dimensional construct mainly composed of collagen fibers. The mean single and double suture retention loads of the SIS patch were 48.6 ± 5.8 N and 117.9 ± 2.7 N, respectively. The DNA content in the SIS patch was 53.9 ± 10.9 ng/mg dry weight. Both the histological score and ultimate load to failure increased in a time-dependent manner in both groups, with no significant differences between the SIS and FL groups at 12 weeks.

**Conclusion:**

Repair of a large supraspinatus tendon defect using a reinforced, low immunogenic, SIS patch achieves similar effects as autologous FL in a rabbit model. This novel patch might be useful to be employed as a structural tissue replacement in medical activities.

## 1. Introduction

Repair of rotator cuff tears, particularly large to massive tears, is associated with a high failure rate of up to 90% at 3-5 years postoperatively [1]. These postoperative failures are influenced by multiple factors, including the hypovascularity of the rotator cuff, size of the original defect, adipose tissues and retraction of the tendon, and the incurrence of forces in the repair site in the early rehabilitation stage [1, 2]. Advancements in tissue engineering have improved the success rate, but each type of grafting material has shortcomings. Autografts cause damage to the donor site and are mechanically weak, allografts involve disease propagation and supply shortage, and synthetic materials result in foreign body reaction, cytotoxic reactions, and bone erosion [3, 4].

Porcine small intestine submucosa (SIS) is reportedly capable of inducing site-specific remodeling of various connective tissues [5, 6]. Dejardin et al. have already demonstrated that SIS was able to induce regeneration of tendon-like tissues in a dog model [7]. However, issues regarding the mechanical and immunogenic properties of SIS have tempered its further use [8–13]. Augmentation with a SIS patch to enhance the repair of large to massive rotator cuff tears is reportedly associated with poor clinical outcomes [12, 14]. This may be partially explained by the early resorption of SIS, which loses much of its mechanical properties before the regeneration of mechanically sufficient tissues [8, 12]. Augmentation repair is considered to work by protecting the repaired tendon from excess forces on the repair site to improve healing; however, the value of currently available SIS patches such as Restore (DePuy, Warsaw, Indiana) and CuffPatch (Arthrotek, Warsaw, Indiana) lies in their biologic rather than mechanical properties [9, 10, 12]. Additionally, many studies have reported an immunogenic response to the residual DNA in SIS [12, 13, 15, 16]. These two abovementioned reasons (early patch resorption and elicitation of an immunogenic response) have limited the use of SIS as an augmentation graft in rotator cuff repair [8, 12, 14].

In the present study, we aimed to develop a SIS patch with strong mechanical properties and low DNA content to “bridge,” rather than “augment,” a tendinous defect in a rabbit model of rotator cuff tear. As autologous fascia lata (FL) has already achieved good results in rotator cuff repair in the clinical setting [4, 17, 18], we aimed to compare the histological and biomechanical results of the SIS repair with that of the FL repair. We hypothesized that the histological and biomechanical properties of regenerated tissues in the SIS group would be comparable with those in the FL group, and this novel SIS patch could be served as a structural tissue replacement in rotator cuff repair model.

## 2. Materials and Methods

### 2.1. Preparation of the SIS Patch

The raw materials were derived from porcine small intestine from specific pathogen-free pigs. The tunica submucosa was obtained after removal of the mucosal, serosal, and muscular layers. To reduce the immunogenicity of the product, sodium hydroxide and sodium chloride solutions with different concentration gradients were used to remove the cellular and DNA components, respectively.

Each single sheet of SIS was approximately 10 *μ*m thick after being dried in the oven (GZX-550ASB, Ke Wei Yong Xing Instrument, Beijing, China) at 37°C until the moisture content was about 50%. To reinforce the mechanical properties of the graft, 30 individual sheets of SIS were laminated together by further 24-hour drying to produce a single non-cross-linked SIS patch, which was approximately 0.5 mm thick. Final sterilization was performed with epoxyethane at 750 mg/L for 6 hours to eliminate the remnant bioburden within layers

### 2.2. Characterization of the SIS Patch

#### 2.2.1. Micromorphology

**Figure 1:**
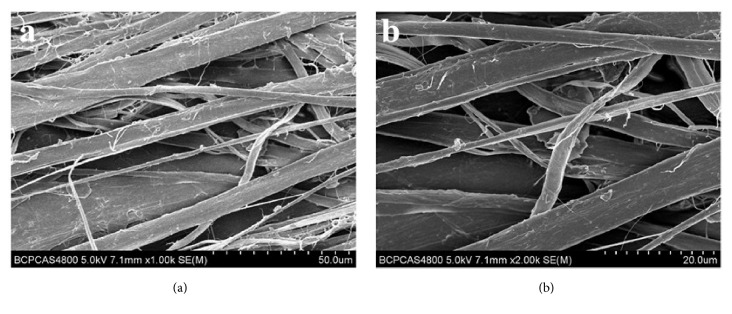
Micromorphology of the SIS patch.

The micromorphology of the SIS patch was evaluated using a scanning electron microscope (FEI Quanta 250, The Netherlands) equipped with a field-emission gun (20 kV) and Robinson detector. Before observation, the sample was gold-coated to minimize the charging effect (Figure 1).

### 2.3. Mechanical Properties

**Figure 2:**
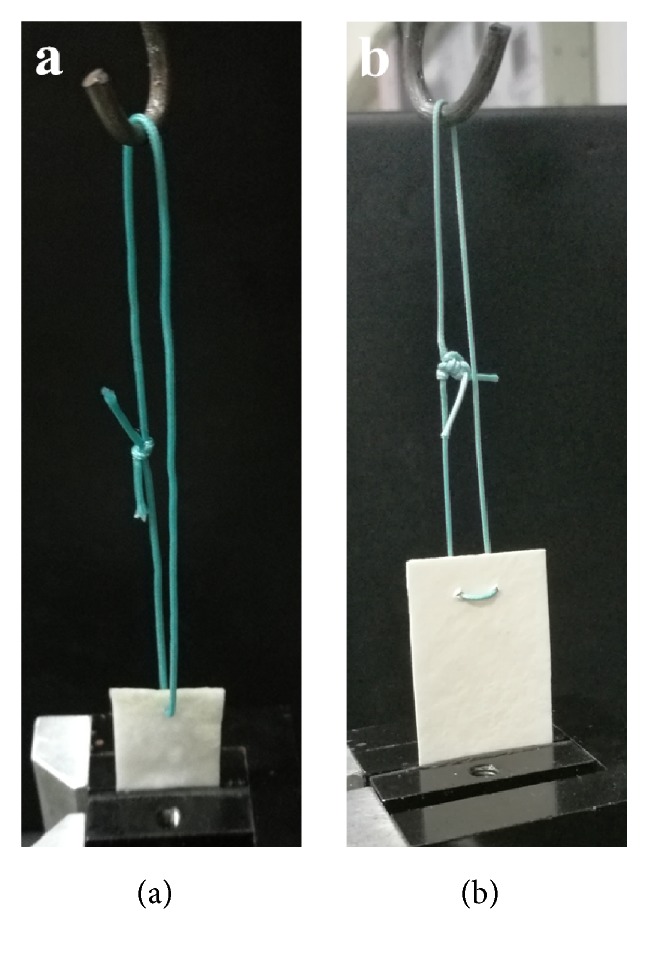
The load-to-failure test of the SIS patch. (a) Single suture retention load test; (b) double suture retention load test.

Each sample was cut into a rectangular strip (2 cm wide and 5 cm long) and soaked in saline gauze for 5 minutes before testing. For the measurement of the single suture retention load, a single suture (polyester, braided, coated, non-absorbable; PremiCron) was placed 5 mm from the 2 cm wide edge of the sample. For the measurement of the double suture retention load, a 5 mm wide horizontal mattress suture was placed 5 mm from the 2 cm wide edge of the sample. The ends of the suture were tied together at the same distance from the sample to the knot. A mechanical testing machine (Instron 5569, USA) was used to conduct the load-to-failure test. The lower half of the sample was fixed in the stationary lower grips with 300 psi compressive pressure to prevent slippage. The tied suture ends were suspended on the “S” hook of the machine (Figure 2). The test began at a distraction rate of 12.5 mm/minute, with the suture ends oriented in the vertical plane. The load to failure was recorded. For both single suture and double suture retention loads, a total of three samples were tested to minimize individual errors.

### 2.4. DNA Content

To quantify the DNA content of the SIS patch, each lyophilized sample was cut into a 50 mg strip, rehydrated in 1 × tris-EDTA buffer solution, and digested with 20 mg/mL of proteinase K (Aladdin, Shanghai Aladdin Biochemical Technology, China) until there was no visible residue. After purification, the DNA content was evaluated using the PicoGreen dsDNA Assay [19]. Briefly, each sample was incubated with or without 50 *μ*g/mL DNase I (Aladdin, Shanghai Aladdin Biochemical Technology, China) and mixed with PicoGreen dsDNA quantitation reagent in 1 × tris-EDTA buffer solution in a 1:1 ratio. After incubation for 10 minutes at room temperature in a dark environment, samples were run continuously in triplicate on a black 96-well assay plate (Molecular Devices, California, USA) at an excitation wavelength of 480 nm and an emission wavelength of 520 nm. The absorbance reading of the DNase-treated sample was subtracted from that of the nontreated sample to correct for background autofluorescence. A total of three tests were conducted to minimize individual errors.

### 2.5. Animal Experimental Design

The experimental protocol was approved by the Animal Care and Use Committee of Shanghai Sixth People's Hospital.

One of the bilateral shoulders was randomly chosen to undergo repair with a SIS patch (SIS group), while the other underwent repair with autologous FL (FL group). The supraspinatus tendon was resected, and a tendinous defect was created (as described below). In the SIS group, the SIS patch was transplanted to repair the tendinous defect between the supraspinatus and the greater tuberosity; in the FL group, the defect was filled with the autologous FL. A power analysis was first conducted to calculate the sample size required for the animal experiments. With an *α* of 0.05, 1-*β* of 0.8, and dropout rate of 25%, a sample size of eight shoulders in each group was required to detect a significant difference in the ultimate load to failure in accordance with a previous study [20]. Hence, the present study included 36 male New Zealand White rabbits with a mean age of 16 weeks and mean weight of 2.5 kg. The histological analysis was performed on four shoulders from each group at 4, 8, and 12 weeks; the biomechanical tests were performed on eight shoulders from each group at 4, 8, and 12 weeks.

### 2.6. Surgical Procedures

**Figure 3:**
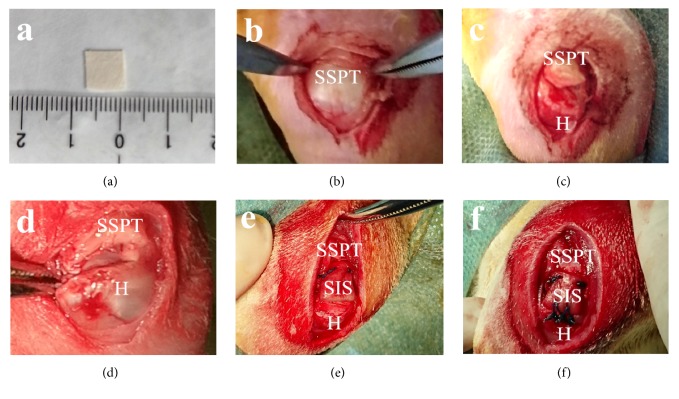
Surgical procedures carried out in the SIS group. (a) Preparation of the SIS patch. (b) Exposure of the supraspinatus tendon (SSPT). (c) Resection of the supraspinatus tendon (SSPT) from the humerus (H). (d) Establishment of an irreparable model after creation of a 5 mm × 5 mm tendinous defect. (e) Fixation of the SIS patch to the remained proximal supraspinatus tendon (SSPT). (f) Fixation of the SIS patch to the humerus (H).

**Figure 4:**
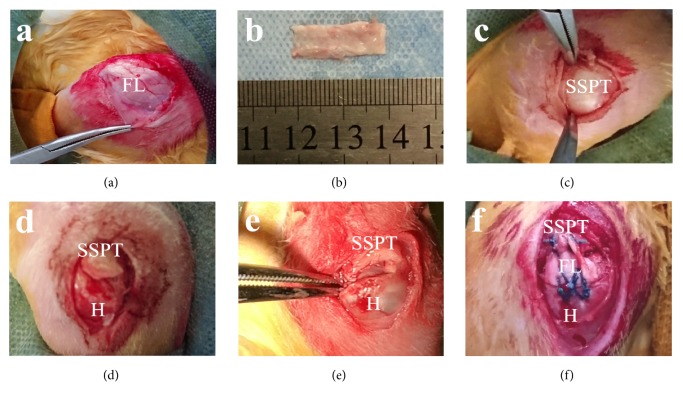
Surgical procedures carried out in the FL group. (a) Exposure of the FL. (b) Harvesting of the FL. (c) Exposure of the supraspinatus tendon (SSPT). (d) Resection of the supraspinatus tendon (SSPT) from the humerus (H). (e) Establishment of an irreparable model after creation of a 5 mm × 5 mm tendinous defect. (f) Grafting of the FL to repair the defect.

Rabbits were anesthetized with intravenous pentobarbital, and the bilateral shoulders were shaved and surgically prepared. Procedures were carried out under sterile conditions. A 10 mm × 10 mm SIS patch was prepared and rehydrated in saline solution for 5 minutes until the time of grafting. To obtain the autologous FL, a 5 cm longitudinal skin incision was made at the lateral aspect of the thigh. An FL strip of 20 mm × 10 mm was harvested and then soaked in saline solution until the time of grafting. A 2 cm longitudinal skin incision was made over the shoulder joint, and blunt dissection was performed to retract the deltoid muscle to expose the supraspinatus tendon. The supraspinatus tendon was sharply resected at the osteotendinous junction, and an irreparable model was established by the creation of a 10 mm × 10 mm full-thickness tendon defect. The greater tuberosity was decorticated to create a bleeding surface, and two bone tunnels were drilled at the original tendon insertion and traveled in parallel to exit at the lateral aspect of the proximal humerus. In the SIS group, the remaining proximal supraspinatus tendon was fixed to the SIS patch using two interrupted sutures (2-0 Ethibond, Ethicon, Germany). The lateral edge of the SIS patch was anchored to the proximal humerus by two parallel transosseous sutures and two crossed transosseous sutures (Figure 3). In the FL group, the FL was first folded to reinforce its mechanical properties; the folded FL was then grafted in an identical manner as the SIS patch (Figure 4). The muscle, subcutaneous tissue, and skin were closed as separate layers.

Rabbits were returned to their cages and monitored until recovery. Each rabbit was evaluated daily regarding appetite, activity, and infection or dehiscence of the surgical sites. Normal cage activities were allowed without immobilization. Each rabbit received intramuscular Penicillin for at least 3 days postoperatively.

At each determined time point, rabbits were euthanized with an intravenous overdose injection of pentobarbital. Histological analysis and biomechanical tests were performed after macroscopic evaluation of the repaired shoulder.

### 2.7. Histological Analysis

Specimens were fixed in 4% paraformaldehyde (PH = 7.4) for 24 hours, decalcified with 0.25 mol/L ETDA in phosphate-buffered saline (PH = 7.6) for 4 weeks, and embedded in paraffin. Consecutive 5 *μ*m thick sections were cut along the long axis of the supraspinatus tendon in the coronal plane. Sections were stained with hematoxylin and eosin, safranin O, and picrosirius red following routine protocols. We employed the tendon maturing scoring system proposed by Ide et al. [21] to semiquantitatively evaluate the regenerated tissues and the tendon-to-bone insertion (Table 1). This semiquantitative histological scoring system included seven parameters: cellularity, proportion of cells resembling tenocytes, the proportion of parallel cells, vascularity, proportion of fibers with a large diameter characteristic of mature tendon fibers, parallel fibers, and tendon-to-bone insertion remodeling. Each parameter was, respectively, evaluated and scored. The final sum of the scores was obtained, with a score of 28 indicating a perfectly arranged tendon, and 7 indicating a maximally abnormal tendon. To avoid subjective bias, all examinations were performed by two independent investigators who were not otherwise involved in the study.

### 2.8. Biomechanical Testing

**Figure 5:**
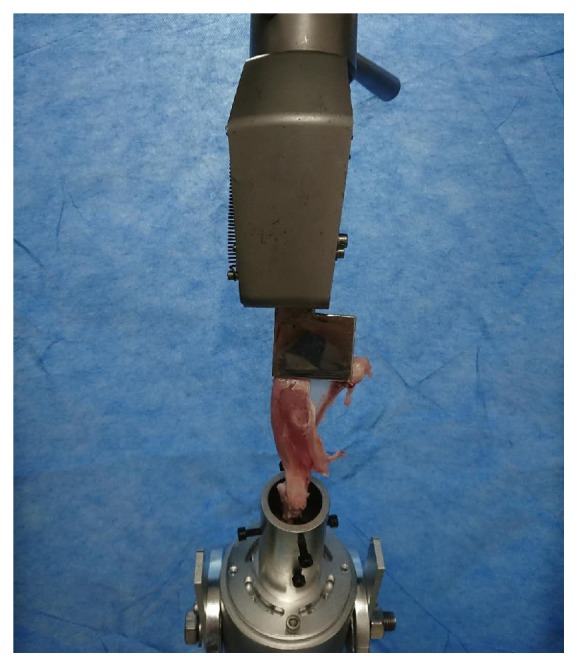
Biomechanical testing of the specimen.

Biomechanical tests were commenced immediately after the harvesting of the specimen, which included the humerus, regenerated tendinous tissues, supraspinatus muscle, and scapula. Sutures were removed before testing. Biomechanical testing was conducted using a machine (Instron 5569, USA) composed of a custom-designed fixture system and a tensile sensor system. The humerus was fixed in a cylindrical holder by six interference screws, and the scapula was fixed in a clamping device connected to the sensor, with the tendinous portion at an angle of approximately 135° to the humerus to mimic the anatomic direction of tensile force (Figure 5). After being subjected to a static preload of 0.5 N for 5 minutes, the specimen received 10 cyclic loads ranging from 5 N to 20 N to minimize the viscoelastic effects. The load-to-failure test was subsequently performed with uniaxial tension at 1 mm/minute. The ultimate load to failure was defined as the first significant decrease in the load-displacement curve. The mode of failure during the test was also recorded.

### 2.9. Statistical Analysis

SPSS software (version 15.0, SPSS Inc, Chicago, Illinois) was used for statistical analyses. In each group, an analysis of variance was first performed using the F test to detect whether there was a significant difference between the inter-time point variations and the intra-time point variations regarding the variables. When the F test indicated the existence of significant differences, the Newman-Keuls q test was used to perform multiple comparisons between the variables from different groups. The significance level was set at 0.05 for the analysis of variance analyses.

## 3. Results

### 3.1. Characterization of the SIS Patch

#### 3.1.1. Mechanical Properties

The mean single suture retention load was 48.6 ± 5.8 N, and the mean double sutures retention load was 117.9 ± 2.7 N. There was no failure of the suture during either single or double suture retention load testing.

### 3.2. DNA Content

The mean DNA content in the SIS patch was 53.9 ± 7.9 ng/mg dry weight.

### 3.3. Animal Experiments

#### 3.3.1. Macroscopic Evaluation

**Figure 6:**
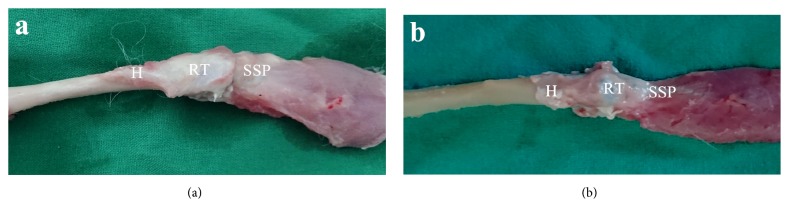
Macroscopic evaluation of the specimen at 12 weeks. (a) Representative specimen from the SIS group at 12 weeks. (b) Representative specimen from the FL group at 12 weeks. SSP: supraspinatus; RT: regenerated tissues; H: humerus.

There was no evidence of infection or foreign body reaction in the SIS, and FL groups. At each harvest time point, the graft was completely surrounded by regenerated tissues. The gross appearance of the SIS patch still could not be discerned until 12 weeks. In both groups, the regenerated tissues showed good continuity and solid integration to the proximal supraspinatus tendon and the greater tubercle (Figure 6).

### 3.4. Histological Analysis

At 4 weeks, both groups showed hypercellularity and hypervascularity. There were many oval- to spindle-shaped fibroblasts, accompanied by newly formed fibers. Fibroblasts and fibers appeared poorly organized. The regenerated tissues showed good continuity to the bone, but were not anchored to the bone. In the SIS group, there was no evidence of formation of positively stained collagen fibers, while in the FL group, there were a few red-stained type III collagen fibers according to picrosirius red stained tissue sections. The interface between the proximal supraspinatus tendon and the graft was fibrously connected in both groups.

At 8 weeks, differences between the two groups were quite enormous. The cellularity and vascularity were markedly reduced in the FL group, but were still obvious in the SIS group. The arrangement of fibroblasts and fibers in the FL group was more orderly organized than that in the SIS group. The amount of red-stained type III collagen fibers was quite similar between the two groups and was increased compared with that at 4 weeks. The red-stained type III collagen fibers were well anchored to the bone; and new chondrocyte formation was observed in the FL group while not in the SIS group. The fibrous connection between the proximal supraspinatus tendon and the graft did not change much compared with that at 4 weeks.

**Figure 7:**
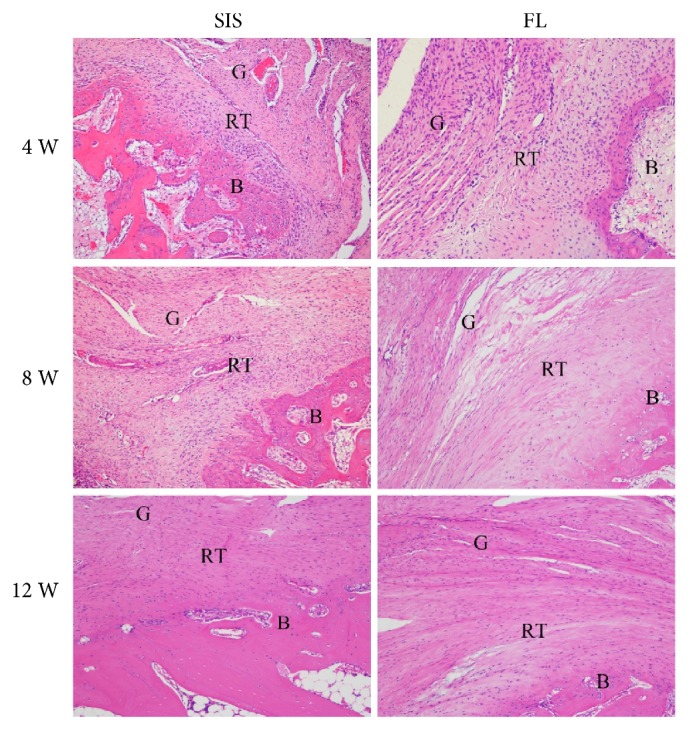
Representative hematoxylin and eosin stained tissue sections of the regenerated tissues and tendon-to-bone insertion at 4, 8, and 12 weeks. G: graft; RT: regenerated tissues; B: bone. 100× magnification.

**Figure 8:**
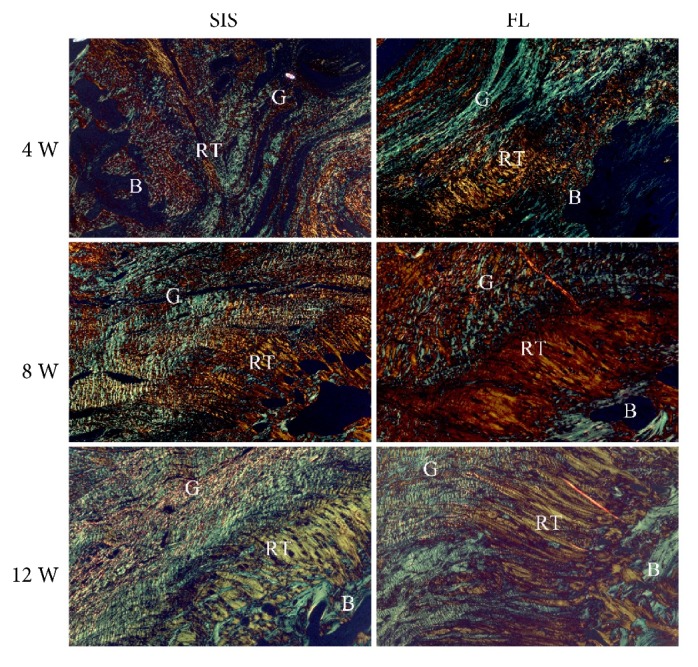
Representative picrosirius red stained tissue sections of the regenerated tissues, and tendon-to-bone insertion at 4, 8, and 12 weeks. G: graft; RT: regenerated tissues; B: bone. 100× magnification.

**Figure 9:**
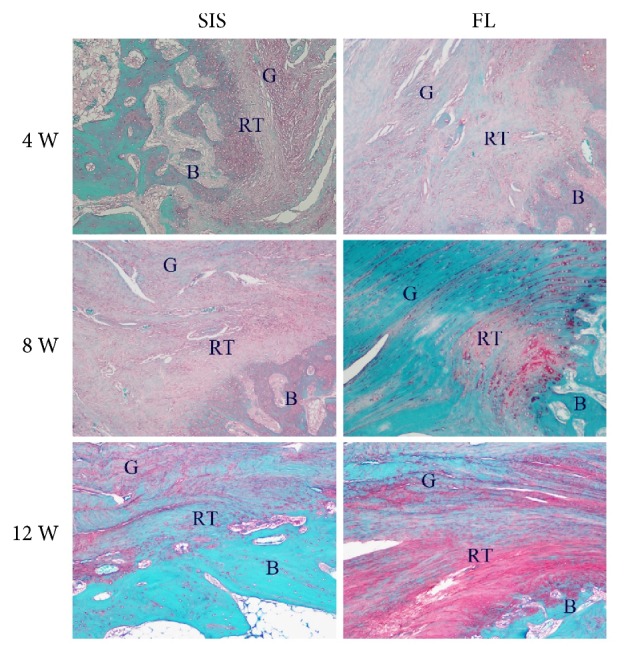
Representative safranin O stained tissue sections of the tendon-to-bone insertion at 4, 8, and 12 weeks. G: graft; RT: regenerated tissues; B: bone. 100× magnification.

At 12 weeks, the graft in the SIS group began to be absorbed, and there were no longer substantial differences between the two groups. The cellularity and vascularity were moderate in both groups. Fibroblasts and the extracellular matrix were arranged in columns. Red-stained type III collagen fibers were abundant and were organized along the longitudinal axis of the tensile force. New chondrocyte formation could be observed in both groups, but there was still no evidence of formation of a tidemark gradually changing from tendon to bone [17]. The regenerated tissues still emerged with the proximal supraspinatus tendon through multiple fibrous sites in both groups (Figures 7–9).

### 3.5. Semiquantitative Histological Score

**Figure 10:**
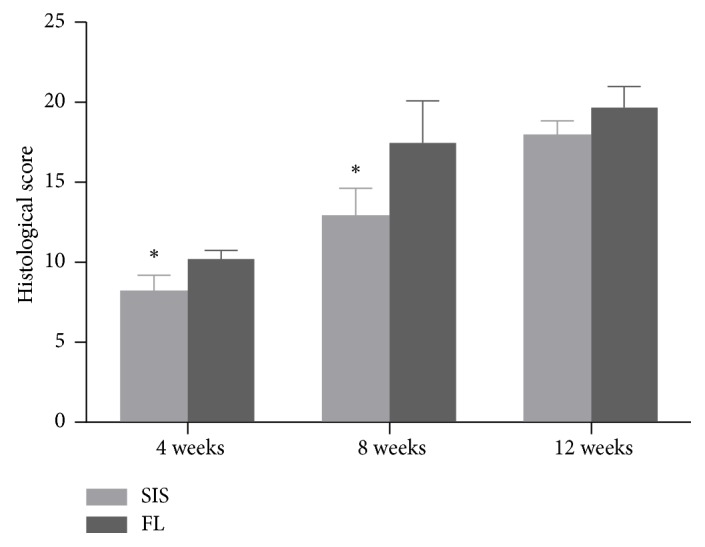
Semiquantitative histological scores. Values are shown as the mean ± standard deviation. *∗* indicates significant differences.

The histological score in both groups increased in a time-dependent manner. At 4 and 8 weeks, the scoring of the SIS group was significantly lower than that of the FL group; however, at 12 weeks, there were no longer significant differences between the two groups (4 weeks: 8.3 ± 1.0 [SIS], 10.3 ± 0.5 [FL], P = 0.01 < 0.05; 8 weeks: 13.0 ± 1.6 [SIS], 17.5 ± 2.6 [FL], P = 0.03 < 0.05; 12 weeks: 18.0 ± 0.8 [SIS], 19.8 ± 1.3 [FL], P = 0.06 > 0.05) (Figure 10).

### 3.6. Biomechanical Testing

**Figure 11:**
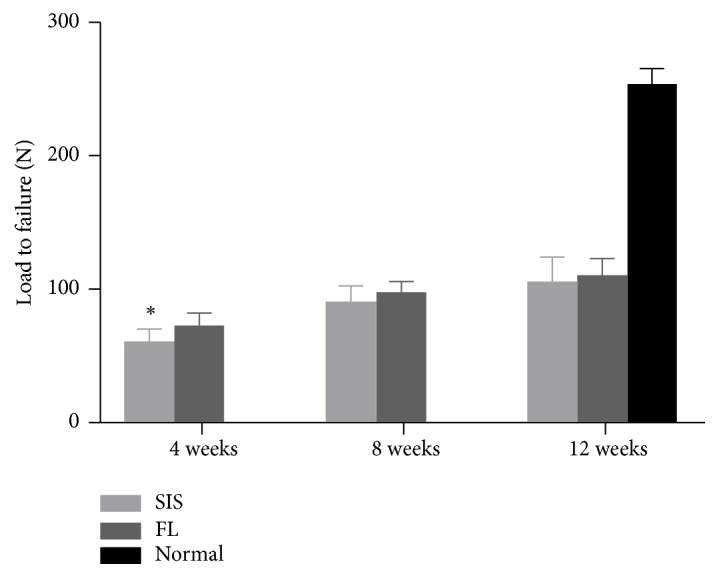
Results of the biomechanical testing. Values are shown as the mean ± standard deviation. *∗* indicates significant differences.

The ultimate load to failure in both groups increased in a time-dependent manner. At 4 weeks, the ultimate load to failure in the SIS group was significantly lower than that in the FL group; however, at 8 and 12 weeks, there were no longer significant differences between the two groups (4 weeks: 60.4 ± 8.8 N [SIS], 73.1 ± 8.6 N [FL], P = 0.01 < 0.05; 8 weeks: 91.0 ± 11.1 N [SIS], 97.3 ± 7.9 N [FL], P = 0.22 > 0.05; 12 weeks: 105.6 ± 18.2 N [SIS], 110.4 ± 12.2 N [FL], P = 0.55 > 0.05). At 12 weeks, the ultimate load to failure in the SIS and FL groups accounted for 41.3 % and 43.1 %, respectively, of that of a normal tendon (Figure 11).

All of the specimens failed at the midsubstance of the graft.

## 4. Discussion

Repair of large to massive rotator cuff tears is associated with a high failure rate of up to 90% at 3-5 years postoperatively [1]. To improve the success of large rotator cuff tear repair, advances have been made in expectant treatment, surgical techniques, rehabilitation strategies, and tissue engineering [2, 8]. An interposition graft protects the repair site from excess tension and promotes tendon regeneration by providing a biologic scaffold [7, 8]. Irreparable rotator cuff tears have been commonly reconstructed using autologous FL in clinical practice, with satisfactory outcomes reported [4, 17, 18]. This successful strategy is mainly attributed to the high degree of similarity of the structural and component properties of autologous FL and the rotator cuff [4]. However, the main limitations of autologous FL are its weak mechanical properties and the trauma caused to the donor site. Thus, scholars have been searching for another type of material to substitute for autologous tissues [3, 7–10, 13, 21, 22]. Among a large number of proposed materials, SIS has the advantages of good biocompatibility and abundant resources [2, 5, 7, 23–25]. In the present study, the reinforced, low immunogenic, SIS patch achieved similar macroscopic, histological, and biomechanical results to autologous FL in the repair of a supraspinatus tendon defect in a rabbit mode. From a macroscopic perspective, there was no evidence of infection or foreign body reaction at any time point, and the regenerated tissues showed good continuity and solid integration to the proximal supraspinatus tendon and the greater tubercle. From a histological perspective, the regenerated tissues of the SIS group mimicked those of the FL group at 12 weeks. From a biomechanical perspective, although the ultimate load to failure at 12 weeks of the SIS group was only 41.3 % of that of a normal supraspinatus tendon, the tensile strength of the SIS group increased in a time-dependent manner and was similar to that of the FL group; furthermore, the midsubstance failure mode demonstrated the strong connection of both the proximal rotator cuff graft and graft bone interfaces. In summary, this novel SIS patch could be served as a structural tissue replacement to repair large to massive rotator cuff tears.

The SIS patch is a three-dimensional construct composed of the natural extracellular matrix, which is capable of inducing migration and deposition of host cells. Host cells adhering to SIS regulate angiogenesis and stimulate the production of collagen, which ultimately results in the formation of tendon-like tissues [2, 7]. Previous studies investigating the inducibility of SIS in augmentation and interposition animal models have already confirmed its excellent biologic properties [2, 7].

However, poor outcomes have been reported when SIS is used to augment the repair of rotator cuff tears in clinical practice [12, 14]. Iannotti et al. [12] reported that patients who underwent repair of rotator cuff tear with SIS augmentation tended to have poorer results than those without augmentation. Furthermore, a multicenter, randomized, controlled study comparing the results of open rotator cuff repairs with those of repairs augmented with SIS did not show either higher functional scores or reduced repair failures at 1 year postoperatively [14]. Hence, the use of SIS to augment rotator cuff repairs was not recommended based on its mechanical and immunogenic properties [8, 12, 15, 16, 26–28]. First, SIS undergoes resorption in the early stages and thus loses much of its mechanical properties, which are not strong enough to support an augmentation repair before the regeneration of mechanically sufficient tissues [12]. Furthermore, even the initial mechanical properties of SIS are much weaker than those of dermal and synthetic grafts [8–10, 13, 26]. Second, previous studies have reported that the immunogenic response to the residual DNA in SIS commonly occurs [15, 16, 27]. Grafts containing cellular DNA can provoke a vigorous immunogenic response, which leads to the formation of scar tissues rather than a biologic matrix at the repair site [28].

In the SIS patch used in the present study, the major weaknesses reported in earlier studies have been improved. The mechanical properties of the present SIS patch were much stronger than those reported previously [9, 10]. The single suture retention load of Restore (DePuy) is reportedly 7.09 ± 1.5 N [10], whereas that of the present SIS patch was 48.6 ± 5.8 N. The double suture retention loads of CuffPatch (Arthrotek, Warsaw, Indiana) and Restore (DePuy) are reportedly 32 ± 1.4 N and 38.2 ± 2.8 N, respectively [9], compared with 117.9 ± 2.7 N for the present SIS patch. The major reason for these discrepancies was that each SIS patch used in the present study comprised 30 single sheets, while CuffPatch and Restore only contained 8 and 10 single sheets, respectively.

The immunogenic response to the present SIS patch may also be decreased, as the DNA content was lower than that of the Restore patch [13]. The DNA content of Restore is 526.8 ± 125.6 ng/mg dry weight [13], while the DNA content of the SIS patch used in the present study was 53.9 ± 7.9 ng/mg dry weight; the other type of SIS patch (CuffPatch) reportedly contains negligible amounts of DNA [13]. It is unknown whether any highly effective acellularization protocol is used for CuffPatch, as there are no specific descriptions concerning this protocol available in the literature. However, the SIS patch used in the present study also had a lower DNA content than other available types of acellularized patches (794.6 ± 97.8 ng/mg dry weight for TissueMend (TEI, Boston, Massachusetts, licensed to Stryker, Mahwah, New Jersey) and 134.6 ± 44.0 ng/mg dry weight for GraftJacket (LifeCell, Branchburg, New Jersey, licensed to Wright, Arlington, Tennessee))[13]. Considering the SIS patch used in the present study not only retained the biologic advantages but had improved mechanical and immunogenic properties compared with previous SIS patches, it is worth investigating whether this type of SIS patch can improve the clinical outcomes in repairs of large to massive rotator cuff tears.

Repair of rotator cuff tears via the application of another types of materials, such as reinforced allogeneic FL, homogenous or heterogenic acellular dermal matrix (ADM), bovine pericardial tissue, and degradable or nondegradable synthetic materials, has also achieved satisfactory results [3, 4, 8, 21, 22, 28]. Poly-L-lactic-acid-reinforced FL is a semisynthetic material that was developed with the aim of improving the poor mechanical properties of FL alone. This type of reinforced FL reportedly has improved mechanical properties at time 0; however, the mechanical properties of the repair are essentially similar to autologous FL at 12 weeks, and a host tissue response was observed [4]. ADM is mainly composed of intact collagens with low immunogenicity and is a widely used patch that is approved by the US Food and Drug Administration [8, 21, 22, 28]. ADM is a good in situ tissue-induced material that has demonstrated strong mechanical properties [5, 9, 10, 13, 21, 22, 25]. Several recent clinical studies found that patients undergoing treatment with either augmentation or interposition repairs with ADM for large to massive rotator cuff tears have significant improvement in pain, range of motion, manual muscle strength, and subjective functional scores [8, 22, 28]. Synthetic materials for rotator cuff tear repair can be made from various compounds such as polyester, polypropylene, polyacrylamide, Dacron, carbon, silicon, or nylon [18]. Although synthetic materials have much stronger mechanical properties than biologic materials, the biocompatibility of synthetic materials is of great concern [18]. Animal studies investigating the use of synthetic materials to repair rotator cuff tears have commonly reported foreign body reaction, cytotoxic reaction, and bone erosion [3, 4], while few human studies have investigated the efficacy of synthetic materials in large to massive rotator cuff repairs [18].

Limitations in the present study lie in the following points. First, the present study used an acute tear model, rather than a chronic tear model. As chronic rotator cuff tears commonly involve adipose tissue and retraction of the torn tendon, the outcomes may differ from those in the acute tear model. The limitation of all extracellular matrix derived devices for rotator cuff repair is that they were validated in acute tear models that do not adequately represent the predominant clinical problem of chronic rotator cuff repair. Anecdotally, the Restore and CuffPatch devices worked well in patients suffering acute tears, but the deleterious tissue changes after a chronic tear make a "regenerative" repair challenging. This was the likely reason for failure of Restore in the Iannotti article, not the strength. Second, although we used the timescale employed by Lewis et al. [29], a longer observation period might be required, as the 12-week time point is most susceptible to mechanical failure during early postoperative rehabilitation. Third, we only evaluated the effect of the SIS patch on interposition models; further studies are needed to assess the effectiveness of this type of SIS patch in augmentation models. Fourth, the properties of the rotator cuff and other conditions in rabbits differ from those in humans, so our results should be considered cautiously before clinical application.

## 5. Conclusion

The novel, reinforced, low immunogenic SIS patch improves tendon regeneration and maturation and achieves similar effects to autologous FL in rotator cuff repair.

## Figures and Tables

**Table 1:** The tendon maturing scoring system.

	1	2	3	4

Cellularity	Marked	Moderate	Mild	Minimal
Proportion of cells resembling tenocytes	<25%	25–50%	50–75%	>75%
Proportion of parallel cells	<25%	25–50%	50–75%	>75%
Vascularity	Marked	Moderate	Mild	Minimal
Proportion of fibers of large diameter characteristic of mature tendon fibers	<25%	25–50%	50–75%	>75%
Proportion of parallel fibers	<25%	25–50%	50–75%	>75%
Remodeling of tendon-to-bone insertion	C(+) I(_)	C(+) I(+) F(_)	C(+) I(+) F(+) T(_)	C(+) I(+) F(+) T(+)

C: continuity; I: ingrowth; F: fibrocartilage; T: tidemark.

## Data Availability

The data used to support the findings of this study are available from the corresponding author upon request.
